# The Role of Task-Related Learned Representations in Explaining Asymmetries in Task Switching

**DOI:** 10.1371/journal.pone.0061729

**Published:** 2013-04-16

**Authors:** Ayla Barutchu, Stefanie I. Becker, Olivia Carter, Robert Hester, Neil L. Levy

**Affiliations:** 1 Florey Institute of Neuroscience and Mental Health, The University of Melbourne, Melbourne, Victoria, Australia; 2 School of Psychology, University of Queensland, Brisbane, Queensland, Australia; 3 Melbourne School of Psychological Sciences, The University of Melbourne, Melbourne, Victoria, Australia; 4 Oxford Centre for Neuroethics, The University of Oxford, Oxford, United Kingdom; University of Muenster, Germany

## Abstract

Task switch costs often show an asymmetry, with switch costs being larger when switching from a difficult task to an easier task. This asymmetry has been explained by difficult tasks being represented more strongly and consequently requiring more inhibition prior to switching to the easier task. The present study shows that switch cost asymmetries observed in arithmetic tasks (addition vs. subtraction) do not depend on task difficulty: Switch costs of similar magnitudes were obtained when participants were presented with unsolvable pseudo-equations that did not differ in task difficulty. Further experiments showed that neither task switch costs nor switch cost asymmetries were due to perceptual factors (e.g., perceptual priming effects). These findings suggest that asymmetrical switch costs can be brought about by the association of some tasks with greater difficulty than others. Moreover, the finding that asymmetrical switch costs were observed (1) in the absence of a task switch proper and (2) without differences in task difficulty, suggests that present theories of task switch costs and switch cost asymmetries are in important ways incomplete and need to be modified.

## Introduction

The ability to switch between tasks rapidly and efficiently affords much flexibility, and facilitates adaptation to environmental or situational needs (e.g., while shopping for groceries switching to attend to an unexpected business call or your child’s needs). However, such task switching comes at a cost. People are significantly better and faster at performing tasks under repeat conditions than when switching between tasks even when tasks are well practiced and people are given sufficient time to prepare for the switch (reviewed in [1]). It is also well known that task switching costs are not symmetrical when switching between tasks of unequal difficulty: Somewhat perplexingly, switch costs are generally greater when switching from a difficult to an easy task than vice-versa. For example, bilinguals have been shown to be slower at switching to their dominant first language than to their secondary language [2], [3], and despite people being faster at reading than naming the colour of incongruently coloured words in a Stroop task, they tend to show greater costs when switching to reading than colour naming [4]–[6]. Similar asymmetries in switching costs have been observed across a range of tasks [7]–[12].

Most models of task switching have relied on task priming effects and top-down controlled inputs to explain task switching effects and the associated asymmetries in switching costs (e.g., [5], [13]–[17]). For example, Allport and colleagues (1994) suggested that the asymmetry in switch costs is due to differences in priming, with difficult tasks leading to stronger positive priming effects and relatively easy tasks being inhibited via negative priming [4], [5]. Using Stroop stimuli, Allport and colleagues [5] also showed that task switching can have long lasting effects and can carry over across several blocks that do not involve task switching. They suggested that priming effects that result in asymmetrical switch costs are the result of learning and the retrieval of conflicting stimulus-response associations from memory (i.e., difficult task are easier to retrieve from memory because of their relatively stronger priming effects). However, the idea of inhibitory priming during task switching is a topic of controversy (e.g., [12], [13], [18]). Yeung and Monsell [13] showed that asymmetries in switch costs can be minimised or reversed by reducing the level of interference between tasks. For example, in a Stroop switch task, when interference in stimulus attributes was minimised (i.e., words were written in black ink on coloured rectangles, and word onset was delayed to make it easier to select task relevant stimuli), not only was the magnitude of the switch costs reduced but the asymmetry was actually reversed; delayed word onset resulted in greater switch costs for colour naming than word reading. Thus, it is not always the case that it is harder to switch to easier/dominant tasks, suggesting that inhibition and negative priming are not automatically engaged during the easier tasks. Yeung and Monsell [13] used a mathematical model to demonstrate that the asymmetries in switching costs can be predicted by considering positive task priming, the strength of activation and the degree of control input. Task priming represents the degree of increased activation by the competing task, while strength of activation depends on the degree of practice or experience with tasks that determines the strength of stimulus-response mappings. Control inputs, on the other hand, refer to top-down inputs applied to minimise bias so that the task can be performed with reasonable accuracy. Indeed the model yielded qualitatively comparable patterns of results for a variety of parameter values, suggesting that priming, prior experience/practice, and top-down control inputs all contribute to switch costs and can explain switch cost asymmetries.

That said and apart from these considerations there is evidence to suggest that asymmetries observed in task switching may not be dependent on task switching per se. Similar performance costs have been observed following a delay or an interruption in the absence of a task-switch, with larger restart costs for easy trials [19]–[21]. These results suggest that asymmetries may partly arise due to differences in information retrieval rates from long-term memory traces rather than interference carryover effects from preceding tasks. More recently, Schneider and Anderson [7] have also shown that switching between different tasks is not essential to induce asymmetric costs. They used vertical and horizontal versions of addition and subtraction equations (with horizontal and subtraction problems being more difficult than additions and vertical equations), to demonstrate that a switch in the orientation of the equation from vertical to horizontal can induce similar asymmetries: When switching between addition and subtraction operations, greater costs were observed for switches to vertical (easy) than to horizontal (difficult) equations. Their proposed *sequential difficulty* account dissociates task-related and difficulty-related switches, suggesting that sequential changes in difficulty levels lead to the depletion of executive control and working memory resources, with less resources being available for an easier task that follows a difficult task, which results in a longer recovery time. Changes in task difficulty may indeed contribute independently to asymmetrical task switching costs; however, the sequential difficulty hypothesis cannot account for residual asymmetries in switch costs observed across blocks and long inter-stimulus intervals (e.g., [5], [19]–[21]), as such effects should dissipate given enough time for recovery under the sequential difficulty account.

Besides task difficulty, there is also evidence to suggest that stimuli used to cue or indicate a task switch, particularly those with strong task related associations, can influence task switching processes (e.g., [22], [23]–[28]). Tasks that are learned via instruction or trial and error are stored in memory, and tasks that are well practiced are easier to retrieve [1]. Such retrieval can be automatically initiated by stimuli associated with specific tasks in the absence of intention to perform a task. Even in situations where one’s intention directly opposes an irrelevant task, it can be difficult to inhibit the task associated with particular stimuli, as shown, for example, in the Stroop effect (i.e., difficulty suppressing word reading when one’s intention is to name the colour). Indeed cognitive task performance is dependent on a complex interplay between endogenously determined goals and intentions, and exogenous factors associated with stimulus properties and context. This interrelationship between cue/stimulus encoding and task performance is further complicated by studies showing that changing between different stimuli can cause switch costs at a perceptual level in the absence of a task switch. For example, during visual search tasks reaction times are slower when stimulus properties of the search target change compared to the preceding trial, than when they are repeated (e.g., [29]). Across a range of tasks, reaction time costs have been observed for changes in both task-relevant and task-irrelevant features of the stimuli [29]–[32], as well as for changes of the stimulus dimension [33]–[37]. However, the interplay between switch costs associated with stimulus/cue perception and competing tasks remains elusive. In particular, how top-down inputs determined by prior environmental learning of associations between stimuli and tasks influence switch costs at a perceptual level is unknown.

## Experiment 1

In this experiment, we investigated top-down influences of learning and experience on switch costs for tasks of unequal difficulty. Prior models have suggested that asymmetries in task switching costs can be partly attributed to previous experience and learning contributing to the strength of stimulus-response mappings and their retrieval from memory (e.g., [5], [13], [16], [26]). Theoretically, within the predictive bounds of such models, extensive practice would lead to strong associations between the task and the stimulus. Hence, if corresponding memory traces are automatically retrieved and significantly contribute to switch cost asymmetries, analogous asymmetries in switch costs should be observed when switching between task-related stimuli in the absence of a task switch. To test this hypothesis we employed an *arithmetic task*, in which participants had to continually switch between addition and subtraction problems. Additions and subtractions have been extensively used in task-switch studies previously, with studies showing that people are faster and more accurate at solving addition than subtraction equations (e.g., [7], [10], [38], [39]–[42]). Correspondingly, we would expect larger switch costs when switching from the more difficult subtraction task to the easier addition task than vice versa.

Asymmetric switch costs in the arithmetic task were compared to switch costs in a *symbol identification task*, where observers only had to indicate whether the symbol was a ‘+’ or a ‘−’, without carrying out any computations. The operation symbols for addition and subtraction problems are extensively practiced from early childhood. Such lifelong experience is likely to result in strong stimulus-task associations in memory that could be activated by the stimuli (i.e., the operation symbols ‘+’ and ‘−’ in our case) even when the arithmetic tasks are not carried out. This experiment aimed to investigate the contribution of such learned associations to asymmetric switch costs. Assuming task switching cost asymmetries are dependent on prior learning and practice, and given the strength of stimulus and task associations with the ‘+’ and ‘−’ symbols, we hypothesised that the symbol discrimination task will show similar asymmetries when switching from ‘+’ symbols to ‘−’ symbols in the absence of a task switch. Here, and in what follows, we maintain the label ‘task switch costs’ to refer to the RT costs typically found on non-repeat trials – even in the absence of a task switch – to facilitate comparisons to previous results and between the tested conditions.

### Methods

#### Participants

Twenty-six healthy adult volunteers participated in this study (7 males and 19 females, *M* age = 21;6 and *SD* = 3;5 years; months). All participants had normal or corrected to normal vision, normal hearing and no prior history of neurological or psychiatric disorders. Participants were paid $AUD 10 per hour for their time. All participants provided written informed consent. This study was approved by, and strictly adhered to the ethics guidelines of the Human Research Ethics Committee, The University of Melbourne.

#### Stimuli and design

**Figure 1:**
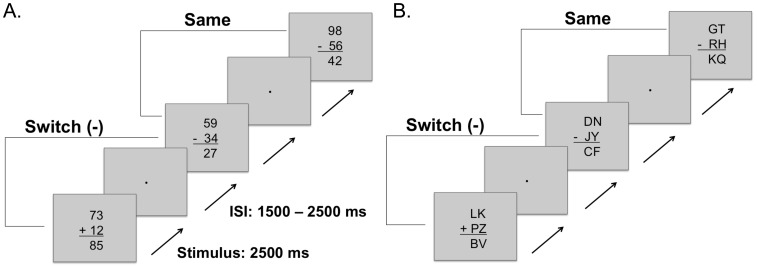
An illustration of the arithmetic switch tasks with solvable and pseudo equations. A. Trials of the arithmetic and symbol-switching task (Experiment 1). For the arithmetic task, participants had to solve equations and indicate whether the answer was correct, while for the symbol-switching task participants had to indicate the type of operation. B. Trials involving pseudo equations of the symbol-switch letter task where participants had to indicate type of operation (Experiment 2).

In Experiment 1 we employed very simple arithmetic tasks with no-carry additions and no-borrow subtractions (e.g., equations that can be solved by adding or subtracting single digit numbers independently of each other and that require no changes in the next (highest) order number; e.g., 12+85 = 97; 76–24 = 52). Equations were presented in the traditional vertical format using the Arial font format and a font size of 24 (see Figure 1A). All three terms of problems were double-digit numbers to allow for the generation of a large sample of unique problems: 992 additions and 1008 subtraction problems. For each participant and condition a new order of equations was generated by randomly selecting from all possible equations. Addition and subtraction problems were randomly correct or incorrect with a probability of.5. Incorrect problems were calculated by randomly adding or subtracting 2 or 9 from the correct answer. Addition and subtraction tasks were randomly chosen on each trial, so that the requirement to switch tasks was unpredictable, requiring participants to prepare for both tasks at the end of each trial (e.g., [2], [43], [44]–[46]). Stimulus duration was 2.5 s with an inter-stimulus interval (ISI) randomly varying between 1.5 s and 2.5 s. The relatively long ISI was used to allow participants to recover from prior trials, prepare for a possible task switch, and to allow for measuring residual switch costs [5], [22], [43].

#### Procedure

Participants were seated in a quiet room at a distance of 70 cm from a 17 inch computer monitor. In the initial practice session, participants were allowed up to 48 trials of practice to familiarise themselves with each task. For the *arithmetic-switching task,* participants were asked to solve the problems and to indicate whether the answer was correct or incorrect by pressing keys with their index and middle finger of their right hands. The alternative *symbol-switching task* was identical to the arithmetic task except that participants were asked to ignore the numbers and the solution to the problems, and indicate the operation type (i.e., discriminate between the ‘+’ and the ‘−’ symbols). Stimulus properties and overall task durations were identical for the arithmetic and the symbol switching tasks. For both the *arithmetic-switching task* and the *symbol-switching task* a random sequences of equations were presented in 4 blocks of 48 trials. The order of the arithmetic- and symbol-switching tasks was counterbalanced across participants.

#### Data analyses

For all analyses the first trial of each block was removed. Only correct responses with reaction times greater than 100 ms and within 4 *SD* above the mean were included in reaction time analyses (less than 1% of trials were excluded based on this criterion – a stricter outlier criterion such as removing RTs above 2.5 *SD* or 3 *SD* of the mean did not change the pattern of results for RTs). This criterion was applied on the mean of each condition and participant. Percent error rates for the arithmetic task were statistically analysed using repeated-measures Analyses of Variance (ANOVA). For all symbol detection tasks, error rates were skewed leading to violations of the assumption of normality. Therefore, for each symbol discrimination task, differences between the four switch and symbol conditions were analysed using Friedman’s Tests followed by post-hoc tests using the Wilcoxon Signed Rank Test. Reaction time data were statistically analysed with repeated-measures ANOVA. All ANOVAs were followed by post-hoc test using the Tukey method where appropriate. This data coding and analysis procedure was applied to all experiments reported in this paper.

### Results and Discussion

A 2(operation type: addition and subtraction)×2(switch condition: repeat and switch) ANOVA computed over the mean error rates of the arithmetic task showed a significant main effect for operation type, *F*(1,25) = 6.72, *p* = .016, *η^2^* = .21, reflecting that overall error rates were significantly higher for subtractions than additions in the arithmetic task (see Table 1). The remaining effects and interactions were all non-significant (main effect for switch condition, i.e., switch or repeat), *F*(1,25) = 1.02, *p* = .32, *η^2^* = .04, and the switch condition×operation type interaction, *F*(1,25) = 0.08, *p* = .78, *η^2^* = .003). When participants were asked only to indicate the type of symbol, error-rates were very low and skewed for all switch conditions leading to large violations of normality (Table 1). The Friedman Test was used to compare the four conditions (i.e., repeat ‘+’, repeat ‘−’, switch to ‘+’ and switch to ‘−’), and showed no significant differences, *χ*2(3) = 1.45, *p* = .695.

**Table 1:** Mean reaction times (RTs; in ms) followed by the Standard Error of the Mean (*SEM*) and mean proportion of errors (ERR, in %) followed by standard deviation (*SD*) for same and switched addition (+) and subtraction (−) conditions, listed separately for the arithmetic task and the symbol-identification task of Experiment 1.

		(+)		(−)	
		Same	Switch	Same	Switch
RTs	Arithmetic	1367±56.29	1423±58.26	1511±57.03	1537±62.42
	Symbol	564±19.04	597±18.74	575±18.45	585±17.57
ERR	Arithmetic	4.26±3.28	4.75±4.02	6.25±5.44	7.07±4.56
	Symbol	1.89±1.87	1.85±2.61	1.83±2.46	2.31±3.21

**Figure 2:**
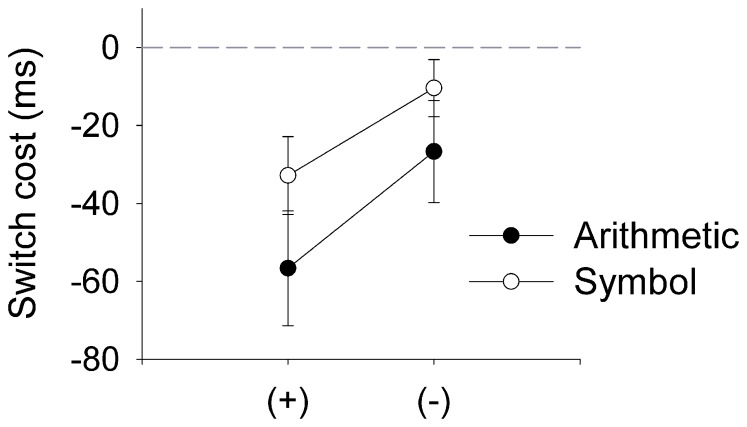
Switch-costs for the arithmetic- and symbol switch task. Reaction time switch costs (computed as RT repeat – RT switch) for Experiment 1, depicted separately for trials that required switching to additions/‘+’ and subtractions/‘−’ and for the arithmetic-switching task (Arithmetic) versus the symbol-switching task (Symbol). Error bars depict the Standard Error of the Mean Difference between repeated and switched trials [66], [67].

Inspection of the reaction times also revealed differences between additions and subtractions for the arithmetic-switching task, however, for both tasks the RTs depended on whether symbols repeated across trials or switched (see Figure 2 and Table 1). Figure 2 shows the ‘switch cost,’ which was calculated by subtracting the RTs for the ‘switch’ conditions from repeat ‘same’ conditions (i.e., switch cost = same − switch). Therefore, a positive value represents a gain in performance from a switch between ‘−’ and ‘+’, while a negative value represents a ‘switch cost.’ Consistent with prior reports, greater costs were observed when switching to an addition than a subtraction task [7]. This pattern of switch cost asymmetry was also observed when participants did not engage in the arithmetic tasks and only reported the type of symbol. Statistical analysis of the data confirmed the impressions: A 2(task type: arithmetic and symbol task)×2(operation symbol: addition and subtraction)×2(switch condition: repeat and switch) ANOVA computed over the mean RT showed a significant main effect for task, *F*(1,25) = 268.14, *p*<.001, *η^2^* = .92, symbol, *F*(1,25) = 34.37, *p*<.001, *η^2^* = .58, and switch condition, *F*(1,25) = 27.18, *p*<.001, *η^2^* = .52. The interaction between task and operation symbol was also significant, *F*(1,25) = 30.43, *p*<.001, *η^2^* = .55. Reaction times were significantly slower for subtraction equations than addition equations in the arithmetic task, but did not differ for the symbol discrimination task. More important for the evaluation of switch cost asymmetries, the interaction between operation symbol and switch condition was significant, *F*(1,25) = 7.53, *p* = .011, *η^2^* = .23. As can be observed in Figure 2, switch costs were significantly greater for switching to an addition than to a subtraction in both the arithmetic and symbol-switching task (Figure 2).

The remaining interactions were all far from significant [task×switch condition interaction, *F*(1,25) = 2.13, *p* = .157, *η^2^* = .08, and the three-way interaction between task, operation type and switch type, *F*(1,25) = 0.12, *p* = .728, *η^2^* = .001].

The finding of similar asymmetrical switch costs in the arithmetic task and the symbol identification task indicates that asymmetries in switch costs do not depend on switching between different arithmetic operations, as the symbol switching task did not include such task switches but only changes of the stimulus (‘+’ vs. ‘−’) and the motor response (left vs right button). Moreover, none of the tasks required changing the stimulus-response associations on switch trials, as the task was to indicate correct versus incorrect results on all trials of the arithmetic task, and to discriminate between ‘+’ and ‘−’ symbols on all trials of the symbol identification task. These facts rule out reconfiguration of stimulus-to-response mappings as a possible explanation for the asymmetric switch costs observed in Experiment 1.

The sequential difficulty account would predict larger switch costs in switching from a more difficult trial to an easier trial, even in the absence of a task switch [7]. However, it is unlikely that the sequential difficulty account can explain the results of Experiment 1. First, responses were equally fast for identifying the ‘+’ and ‘−’ symbols in the symbol identification task (580 ms and 580 ms, respectively). Secondly, because of the relatively long ITI, the time between successive responses was close to 4 seconds in the symbol identification task, and close to 3 seconds in the arithmetic task (on average), rendering it very unlikely that asymmetrical switch costs could be due to greater depletion of executive control and working memory resources in more difficult tasks. It is more likely that the asymmetry in the present study is driven by learned associations with task difficulty (i.e., subtraction being significantly more difficult than additions).

Indeed most models aimed at explaining asymmetries in switch costs suggest that such paradoxical costs are partly related to prior practice and learning, and the rate of retrieval of information from memory, with difficult tasks having stronger associations leading to stronger priming and faster retrieval of information (e.g., [5], [13]). In the context of the present study, asymmetries in switching from ‘−’ symbols to ‘+’ symbols could hence be due to *learned associations*: It is possible that ‘−’ symbols were automatically associated with subtraction tasks, and that switch costs were larger in switching from ‘−’ to ‘+’ symbols because of the *associated difficulty* that in turn affected the ease or speed of retrieving memory traces from the previous trial. In line with this possibility, previous studies indicate that visual stimuli can act like strong mnemonics and trigger memory retrievals [47]–[49]. The ‘−’ symbol being associated with a harder task may cause stronger representations in memory leading to greater activation levels than the ‘+’ symbol; this expectation or knowledge of a subtraction being more difficult than an addition task could drive switch asymmetries in the absence of a task via top-down learned associations.

Alternatively, participants may have been automatically solving equations during the symbol-switching tasks despite being asked to ignore problems and their solutions. Contrary to this possible explanation, reaction times in the symbol-switch task were much faster than for the arithmetic switch task (see Table 1), indicating that participants were not solving the equations during symbol identification. However, stimulus presentation properties were identical between the arithmetic- and symbol-switch tasks, therefore, participants would have had enough time to solve equations after identifying the symbol type.

Experiments 2 and 3 were designed to control for this possibility, as well as other alternative explanations for the outcome of Experiment 1. To ensure that differences between the present results and the control conditions implemented in Experiments 2 and 3 are due to differences in the experimental manipulations and not to individual differences in the tested groups, and to allow for a comparison between the conditions more directly, the same participants who took part in Experiment 1 were re-tested in Experiments 2 and 3.

## Experiment 2

Experiment 2 addressed the possibility that subjects in Experiment 1 may have been solving the equations in the symbol-task by examining whether the observed asymmetry persists with unsolvable pseudo equations. It also addressed the issue of whether stimulus presentation time has an influence on asymmetrical switch costs. Prior studies have shown that stimulus presentation times and intervals between trials can influence the magnitude of the switching cost (e.g., [22], [43], [45], [50]). Also, most prior studies used strategies where stimuli offset upon response (e.g., [7]), rather than being presented for a fixed presentation time as in Experiment 1. We aimed to further investigate the effects of this stimulus presentation strategy on switch cost asymmetries. In our initial experiment, stimuli were presented for the fixed time of 2.5 seconds so the two tasks were identical in all regards exempting only the task instruction. In Experiment 2, we used the symbol-switch task with additional controls (i.e., unsolvable pseudo equations), and also employed an alternative version where stimulus offset was initiated by the participant’s response. In both cases, participants were able to view the stimuli for as long as required to make a response, however the overall task duration was much shortened when stimuli offset upon response.

### Methods

#### Participants

Participants in Experiment 1 were invited back to take part in Experiment 2 (1–3 months after participating in the initial experiment). All participants who took part in Experiment 1 also participated in Experiment 2. Participants were contacted one week prior to testing.

#### Stimuli and procedure

Stimuli and procedure for the *letter symbol-switch task* was the same as in the original symbol-switching task of Experiment 1, where participants responded to the symbols. However, in Experiment 2, the numbers were replaced with capital consonant letters of the Latin alphabet (Figure 1B) on all trials. Consonants were randomly selected from all possibilities, with English words excluded. In two blocks of the task, stimuli were either presented for a fixed duration of 2.5 seconds, as in Experiment 1, or stimulus offset was initiated by the motor response. The order of blocks was counterbalanced across participants.

### Results and Discussion

Participants performed the letter symbol-switch tasks with very high accuracy, thus percent error rates were very low for all conditions (see Table 2). A Friedman Test comparing the error scores in the four switch conditions (i.e., repeat ‘+’, repeat ‘−’, switch to ‘+’ and switch to ‘−’) showed no significant differences for the letter symbol-switch task, *χ*2(3) = 2.64, *p* = .450, or for the letter symbol-switch task when stimuli offset upon response, *χ*2(3) = 5.88, *p* = .118.

**Table 2:** Mean reaction time (RTs; in ms) followed by the Standard Error of the Mean (*SEM*) and mean proportion of errors (ERR; in %) followed by standard deviation (*SD*), depicted separately for same and switched additions (+) and subtractions (−), for the original symbol-switching task (from Experiment 1), the symbol-switching task with letters (from Experiment 2), and the stimulus offset condition from Experiment 2, where the letters offset upon response (Fast Offset).

		(+)		(−)	
		Same	Switch	Same	Switch
RTs	Symbol withnumbers	564±19.04	597±18.74	575±18.45	585±17.57
	Symbol withletters	596±21.88	634±24.28	607±23.93	617±24.98
	Stimulusoffset	519±12.02	545±11.89	520±11.85	539±12.30
ERR	Symbol withnumbers	1.89±1.87	1.85±2.61	1.83±2.56	2.31±3.21
	Symbol withletters	1.72±1.79	1.62±2.24	2.22±2.44	2.76±4.94
	Stimulusoffset	2.70±2.97	2.43±2.61	3.30±3.05	2.53±3.26

**Figure 3:**
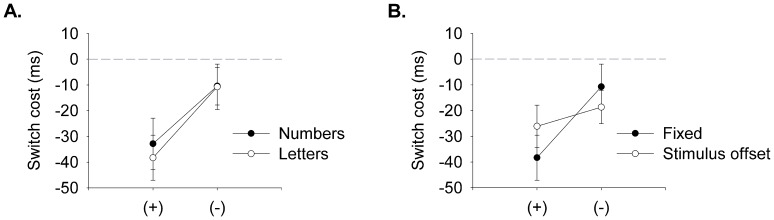
Switch costs for symbol-switch tasks. Reaction time switch costs (computed as RT repeat – RT switch) for **A**. the symbol-switching task using numbers equations (Numbers; from Experiment 1) and letter equations (Letters), and **B**. the symbol-switching task using only letter equations when stimulus presentation is fixed at 2.5 seconds (Fixed) and when stimuli are offset upon motor response (Stimulus offset), from Experiment 2. Error bars depict the Standard Error of the Mean Difference between repeated and switched trials [66], [67].

Inspecting the results in the mean RT revealed that, consistent with the outcomes of Experiment 1, switch costs were significantly greater when switching to an addition than a subtraction symbol (Figure 3A). To examine whether switch costs occurring with unsolvable pseudo-equation differ significantly from the switch costs observed with the symbols in Experiment 1, a repeated-measures ANOVA was computed over the data from the symbol identification task from Experiment 1 and the results of the equivalent condition with unsolvable pseudo-equations of Experiment 2. A 2(task type: symbol task and letter-symbol switch task)×2(symbol: addition and subtraction)×2(switch condition: repeat and switch) ANOVA computed over the mean RT showed only a significant main effect for switch condition, *F*(1,25) = 14.94, *p* = .001, *η^2^* = .37, and the theoretically important interaction between symbol and switch condition, *F*(1,25) = 9.75, *p* = .004, *η^2^* = .28, reflecting the asymmetrical switch costs. The remaining effects and interactions were all non-significant [main effects of symbol type, *F*(1,25) = 0.14, *p* = .711, *η^2^* = .01, task type, *F*(1,25) = 2.21, *p* = .149, *η^2^* = .08, task×symbol type interaction, *F*(1,25) = 0.14, *p* = .708, *η^2^* = .01, task×switch condition interaction, *F*(1,25) = 0.15, *p* = .703, *η^2^* = .01, and the three-way interaction, *F*(1,25) = 0.14, *p* = .711, *η^2^* = .01].

A second repeated-measures ANOVA was computed over the mean RT of Experiment 2, to test whether the stimulus presentation duration affect switch costs and switch costs asymmetries. A 2(task type: letter task and offset task)×2(symbol: addition and subtraction)×2(switch condition: repeat and switch) ANOVA revealed a significant main effect for task, *F*(1,25) = 20.90, *p*<.001, *η^2^* = .46, and switch type, *F*(1,25) = 17.92, *p*<.001, *η^2^* = .42. Reaction times were faster when stimuli offset upon response and for repeat conditions. The interaction between symbol type and switch condition was also significant, *F*(1,25) = 4.90, *p = *.036, *η^2^* = .16. Switching from a subtraction symbol to an addition symbol resulted in a significantly greater cost than vice versa (see Figure 3 and Table 2). No other main or interaction effects approached significance [main effect for symbol type, *F*(1,25) = 0.33, *p = *.571, *η^2^* = .01, task×symbol type interaction, *F*(1,25) = 0.01, *p = *.914, *η^2^*<.001, task×switch condition interaction, *F*(1,25) = 0.15, *p = *.699, *η^2^* = .01, and the three-way interaction, *F*(1,25) = 2.11, *p = *.159, *η^2^* = .08]. Thus, switch costs were significantly greater when switching from ‘−’ to ‘+’ symbols even when equations are unsolvable.

## Experiment 3

Experiment 2 demonstrated that the greater magnitude of observed asymmetry in the switch cost for the ‘+’ in Experiment 1 cannot be attributed to participants solving the equations consciously or sub-consciously. These results suggest that the asymmetry in switching may be driven by learning- and experience-dependent processes, which lead to the retrieval of task related memory traces. However, an alternative explanation for the observed results is that the asymmetry could be determined by perceptual processes. The ‘+’ symbol may be easier to detect due to its greater size, despite the fact that mean accuracy and response times remained unaffected. Hence, switching to a ‘+’ from a ‘−’ symbol may lead to larger switch costs than the reverse, switching from a ‘−’ symbol to a ‘+’ symbol.

Experiment 3 was designed to test whether priming on the perceptual level can account for asymmetrical switch costs. To that end, the ‘+’ and ‘−’ symbols in Experiment 3 were presented in different rotations. If asymmetrical switch costs in the symbol identification tasks of previous experiments were due to differences in perceptual priming, then the same asymmetries would be expected with the rotated symbols. By contrast, if asymmetrical switch costs are due to learned associations of arithmetic symbols, than we would expect no asymmetries in switch costs in Experiment 3, because the rotated symbols cannot be readily associated with arithmetic task and their underlying difficulty.

Experiment 3 also explored possible differences in switch costs arising from differences in size between symbols on successive trials, by presenting the rotated ‘+’ and ‘−’ symbols in two different sizes. The participant’s task was to indicate whether displays contained a single line or two crossed lines.

### Methods

#### Participants

The participants were the same as in Experiments 1 and 2. All participants completed the letter symbol-switching task of Experiment 2 first, followed by a short break before commencing Experiment 3.

#### Stimuli and procedure

The procedure was the same as in Experiment 2 with stimuli offsetting upon participant response. Stimuli consisted of rotated versions of the ‘+’ and ‘−’ symbols. Note that the ‘+’ and ‘−’ symbols, and other rotations that are representative of the division (/) and multiplication (×) symbols were not used, to minimise possible confounds related to learning and experience with these arithmetic symbols. For the ‘+’ symbol 4 rotations were used (15, 30, −15, and −30 degrees) and for the ‘−’ symbol 8 rotations were used (15, 30, 105, 120, −15, −30, −105, and −120), so for both rotated symbols the same angles appear in the original small size (font 24) and the new big (font 48) size. The four rotated stimulus type (crossing-lines small, crossing-lines big, single-line small, single-line big) were presented randomly with equal probability for ‘single’ and ‘crossing lines’ in four blocks of 144 trials. Participants were asked to indicate whether they perceived a single line or two lines crossing with the index and middle fingers of their dominant hand.

### Results and Discussion

Consistent with prior experiments, the percentage of error rates was low (see Table 3). A Friedman Test was used to compare across the 16 conditions (2 symbols×2 sizes×4 switches), which showed only significant differences between the conditions, *χ*2(15) = 27.77, *p* = .023 (note that the Friedman Test is not significant when stricter criteria are employed for defining errors based on RT outlier, i.e., 2.5 *SD* or 3 *SD* above the mean). Post-hoc tests were performed using the Wilcoxon Signed Rank Test (with the large number of comparisons Bonferroni adjustments were deemed to be too conservative; therefore, for post hoc tests the alpha level was adjusted to.01 for the post-hoc tests [51]). The ‘single line’ repeat and switch size conditions significantly differed from the ‘two lines crossing’ switch symbol and switch both size and symbol conditions. Importantly, the 4 repeating conditions for the big and small symbols did not significantly differ from each other, suggesting that difficulty levels did not differ between the symbols and sizes under repeat conditions. Also, when repeat conditions were compared against their respective switch conditions (i.e., switch size, switch symbol and switch both) within each symbol type and size conditions, all of the contrasts failed to reach significance.

**Table 3:** Mean reaction times (RTs, in ms) followed by the Standard Error of the Mean (*SEM*) and mean proportion of errors (ERR) followed by standard deviation (*SD*), for big and small single line or two crossed line stimuli, depicted separately for no switch (Same), switched size, switched symbol and size and symbol switched conditions of Experiment 3.

		Two Lines Crossing		Single Line	
		Big	Small	Big	Small
RTs	Follow same	498±15.56	524±21.66	501±14.12	513±14.28
	Switch Size	500±15.22	525±16.45	504±13.45	516±14.46
	Switch Symbol	544±16.33	551±15.79	533±13.73	542±14.50
	Switch Both	537±14.44	554±15.36	530±14.95	547±14.27
ERR	Same	3.05±2.91	2.36±3.21	2.48±2.94	1.50±1.92
	Switch Size	3.71±3.24	2.53±3.44	3.06±2.81	1.88±2.77
	Switch Symbol	2.82±3.05	4.56±3.75	2.82±2.60	3.03±3.17
	Switch Both	2.61±2.87	3.20±3.32	1.98±2.22	2.77±2.89

**Figure 4:**
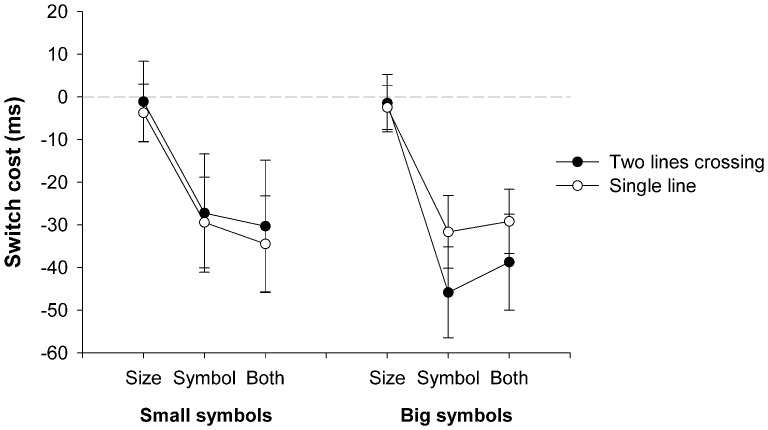
Switch costs for symbols type and size. Switch costs in the mean reaction times (RTs) for Experiment 3, depicted separately for small and big single-line symbols and two-lines crossed symbols when only the size switched, only the symbol switched, or both the size and symbol switched (computed as RT on repeat trials – RT on switch trials). Error bars depict the Standard Error of the Mean Difference between repeated and switched trials [66], [67].

Figure 4 depicts the mean switch costs observed in the reaction times. A 2(symbol type: single line or two lines crossing)×2(size: small and big)×4(switch condition: repeat, switch size, switch symbol, switch both symbol and size) ANOVA computed over the mean RT revealed a significant main effect for size, *F*(1,25) = 65.25, *p*<.001, *η^2^* = .72. Overall RTs were significantly faster for the big symbols than the small symbols (see Table 3). The main effect for switch type was also significant *F*(3,75) = 19.79, *p*<.001, *η^2^* = .44. Switching between different symbols (i.e., from a single line to the crossed lines or vice versa) incurred a significant bi-directional switch costs, whereas a switch in the size of the symbol did not incur any costs (Figure 4). No other main effect or interaction effect approached significance [main effect for symbol, *F*(1,75) = 0.59, *p* = .450, *η^2^* = .02, symbol×size interaction, *F*(1,75) = 2.68, *p* = .114, *η^2^* = .10, symbol×switch type interaction, *F*(3,75) = 0.29, *p* = .833, *η^2^* = .01, size×switch type interaction, *F*(3,75) = 1.37, *p* = .259, *η^2^* = .05, three-way interaction, *F*(3,75) = 0.97, *p* = .411, *η^2^* = .04].

The asymmetry observed in Experiment 1 and 2 for the ‘+’ and ‘−’ symbols was not observed when the small symbols were rotated. With the big symbols, the switch costs tended to be greater when switching to ‘two-lines’ crossing than to a ‘single line’, though this effect was not significant. While this numerical difference is interesting in its own right, it cannot explain the previous results, because the previous experiments all used the small size symbols (24pt), which did not show any signs of asymmetrical switch costs. Hence, the asymmetry in switching from ‘−’ to ‘+’ symbols in the previous experiments cannot be due to switch costs at the level of perceptual processes, but rather arithmetic task-related learned associations of subtractions with greater difficulty than additions.

## General Discussion

The outcomes of these experiments provide new evidence that asymmetrical switch costs generally observed between tasks of unequal difficulty can be induced by task related symbols in the absence of a switch in task and in difficulty. The observed switch asymmetries when solving addition and subtraction problems was also evident when participants switched between the ‘+’ and ‘−’ arithmetic symbols in unsolvable pseudo equations of equal difficulty. The asymmetry in switch costs was eliminated when symbols were rotated so they no longer represent meaningful operations. These findings suggests that switch cost asymmetries may be partly determined by top-down processes related to learned associations attributed to the task related symbols (i.e., subtraction being harder than addition for arithmetic symbols), with such exogenous stimuli leading to the automated retrieval of task related long-term memory traces.

Of note, in the present series of studies, asymmetrical switch costs were observed (1) in an arithmetic switching task, where only the required computation (subtraction vs. addition) changed, whereas the required response (correct vs. incorrect equation) varied independently of these contingencies. Asymmetrical switch costs in the same direction were observed (2) in a symbol identification task, where the ‘+’ and ‘−’ symbols randomly changed together with the required response. Experiment 3 showed that the observed results cannot be explained by switch costs at the level of perceptual processes or response priming, as rotated ‘+’ and ‘−’ symbols in a smaller font size did not show asymmetries in switch costs, even though the stimulus and the required responses changed in the same manner as in Experiment 2. Lines and crosses in a larger font size did show non-significantly greater switch costs in switching from lines to crosses than vice versa. However, the larger font was not used in previous experiments and was only used to explore whether changes from small to large stimuli may be responsible for asymmetrical switch costs. As shown in Figure 4, changing the size of the stimuli did not lead to switch costs and did not modulate switch costs that resulted from switching from lines to crosses (or vice versa). This rules out the possibility that difference in the size of ‘+’ versus ‘−’ symbols played a role in mediating the pattern of switch costs. Given this negative result and the fact that differences in switch costs for the large lines and crosses were also non-significant, it is unlikely that asymmetrical switch costs in the previous experiments were due to processes at the perceptual level.

Perceptual priming or habituation accounts are also unlikely candidates for an explanation of the present findings because they would not predict that switch costs should be larger for switching to a ‘+’ than to a ‘−’ symbol. According to a purely perceptual account, we could expect the reverse asymmetry, with larger costs for ‘−’ trials preceded by ‘+’ trials than vice versa. This holds because the ‘−’ symbol contains only attributes that are a subset of the perceptual attributes of the ‘+’ symbol, whereas the ‘+’ symbol has an additional feature that is not habituated to. Therefore switch cost should be larger for a ‘−’ trial following a ‘+’ trial than vice versa, which is directly contrary to the observed results. Furthermore, in Experiment 3, symbol size did affect reaction times, yet a switch in symbol size had a minimal effect on switch costs. Prior studies have shown that changes in stimulus features and dimensions are more likely to produce switch costs when they are task-relevant [29]–[32], [35], [36], [52]. However, in our case stimulus size was not a feature of attentional focus. Consistent with our initial experiments, motor responses were mapped to symbol type, but not size; participants were asked to make a response to a particular symbol type, therefore, a change in symbol size was not task-related. Taken together with the finding that asymmetrical switch costs were eliminated when the ‘+’ and ‘−’ symbols are rotated and no longer depicted arithmetic operations, the findings suggest that it is the task related associations acquired through learning and experience that are driving the asymmetry in switch costs.

The present findings are important in that they can help elucidate possible sources for the elusive asymmetry in switch costs. Residual asymmetric switch costs persist even when participants are given enough time to prepare for the task switch and have proven difficult to explain by current models for task switching [1], [5], [19], [50], [53]–[55]. According to Rogers and Monsell [22], residual switch costs are due to the fact that people are unable to completely prepare for a switch, and that part of task set reconfiguration occurs only at the onset of the trial, in response to perceiving particular stimulus attributes that signal a particular task. According to De Jong [50], participants can in principle prepare for an upcoming task switch, but due to endogenous factors, task set reconfiguration fails on a portion of trials, for example, because the relevant information cannot be retrieved, so that task set reconfiguration is performed only after the onset of the stimulus. The present findings suggest that another mechanism contributes to asymmetric switch costs; our findings indicated that asymmetric switch costs can occur even when the tasks were equally difficult (i.e., when mean RT to ‘+’ symbols and ‘−’ symbols do not differ; see Table 1), which indicates that automatic retrieval of learned associations about *perceived difficulty* is responsible for the asymmetrical switch costs.

The symbol switching task did not require task set reconfiguration, as the task was always the same; yet, switching from a ‘−’ symbol to a ‘+’ symbol produced higher switch costs than the reverse switch. This result cannot be explained by any of the extant models of task switching and suggests that highly familiar symbols can lead to automatic retrieval of task-irrelevant associations from long-term memory that interfere with the task. This account differs from previous explanations in that it assumes that – even without any need for task set reconfiguration – highly familiar stimuli can automatically trigger retrieval, including retrieval of task-unrelated information about the difficulty of arithmetic tasks, which in turn interferes with responses in the current task.

Asymmetrical switch costs may also be due to a negative memory association with perceived difficulty. In normal adults, associating the ‘self’ with ‘bad’ in an implicit-associative-task can increase switch cost when switching to a neutral task, suggesting that the retrieval of ‘bad’ or negative memories are sufficient to increase switch costs [56], [57]. The ‘−’ symbol may be associated with negative memory traces, compared to the ‘+’ symbol, for instance, because subtraction equations have a higher difficulty level [7], [10], [38]–[42]. According to these accounts, asymmetric switch costs for symbols are in part due to the fact that perception of arithmetic symbols can automatically trigger the retrieval of task-related associations that modulate responses.

The fact that asymmetric switch costs were observed without a true task switch moreover indicate that task-related associations modulate processes at a very general level, not only processes that are specifically involved in task switching (e.g., task set reconfiguration). Depletion of executive control [7] seems unlikely to account for the asymmetric switch costs observed in the present study, as the tasks did not differ in difficulty (see Experiment 2), and shortening the recovery time (in the stimulus offset condition, Exp. 2) did not increase the costs of switching from the 'more difficult' task to the 'easier' task, but non-significantly decreased these switch costs (Figure 3B). However, it cannot be completely ruled out that depletion of executive control contributed to the switch cost asymmetry. Similarly, the results are consistent with other accounts, including proposals that switch costs asymmetries are mediated by stronger priming effects, interference from the previous task, differences in the retrieval of memory traces, or inhibition accounts. Of note, to account for the present results, the proposed mechanisms would have to operate on processes involved in object identification (including retrieval of information that may aid object identification and/or mapping the perceptual input to semantic categories), or response selection (including the maintenance and retrieval of stimulus-response mappings), as the tasks did not involve processes that are otherwise typical of task switching (e.g., task set reconfiguration or task preparation). The results of Experiment 3 ruled out that the switch cost asymmetry was due to purely perceptual processes that either facilitate or hamper responses given stimuli of different complexity (i.e., an asymmetrical feature priming effect). Switch costs have also been observed in other tasks, including object identification tasks that required reconfiguring the stimulus-response mappings of stimuli from different stimulus dimensions [37], [58]–[60], and working memory tasks that required switching between different items in working memory [61]–[64]. Although it is possible that the same processes that caused switch costs in these tasks are also responsible for the switch costs observed in the present study, this is far from certain: Of note, these studies typically either did not analyse the results in a fashion that would allow assessing asymmetries in switch costs, or the results did not show asymmetrical switch costs [37], [58]–[65]. Moreover, these studies often used markedly different experimental paradigms and stimuli, which renders it difficult to compare the results to the results of the present study. Nevertheless, these studies imply that switch costs are not limited to ‘task’ switching and can be induced across other perceptual and cognitive dimensions, such as switching between items in working memory. Further research is needed to establish whether such switch costs are also prone to asymmetries associated with difficulty, learning and experience.

In sum, the experimental paradigm, methods and results of the present study most closely match those of previous studies in the task-switching literature. Given the close resemblance, it is plausible that retrieval of learnt associations modulated task switching costs in previous studies as well and could potentially complement existing explanations of asymmetric task switch costs. That being said, it should also be noted that task switch costs in the present Experiment 1 were larger in the arithmetic task compared to the symbol identification task. Similarly, asymmetries in switch costs were more pronounced in the arithmetic task than the symbol identification task. Although these differences failed to reach significance, they do indicate that automatic retrieval of learned associations is unlikely to account for the totality of switch costs. Rather, processes such as task-set reconfiguration will certainly contribute to task switching costs. Further research is required to investigate the exact contributions of automatic retrieval of learned associations to task switch costs.

The present series of experiments focused on unpredictable switch costs. Predictable sequences do have the advantage of allowing for task preparation, which can reduce switch costs and residual carry over effects across sequential trials [45]. However, the disadvantage with predictable alternating-runs is that participants have to track not only the task but also the sequence of events, and it is assumed that participants are able to follow the sequence of events without losing track. Unpredictable strategies also have their disadvantages, particularly in regard to task preparation. Using a large stimulus interval (>1 second) would allow for effects from preceding trials to dissipate, however participants are unable to prepare for the upcoming trial and are therefore, required to either prepare for both tasks, or to guess and prepare strategies accordingly. Also unpredictable cues and exogenous stimuli could require more attentional resources. Indeed, the analysis and interpretation of stimuli may act like a task in itself [45]. Nevertheless, the unpredictability of stimuli cannot explain the asymmetry in switch cost in our experiments, since all stimuli were presented unpredictably with equal probability. Further research is needed to investigate whether and to what extent learned associations play a role when trials are predictable.

Asymmetrical switch costs are the by-product of a complex relationship between stimuli, learned associations, and response strategies. Indeed, asymmetries in switch cost can be observed in the absence of a task switch given adequate practice suggesting that they are partly driven by prior learned associations. Further research is required to determine how much practice and experience is required to establish learned associations, and for them to dominate performance in stimulus switching tasks. Given the importance of such tasks and the number of which are over learned (e.g., arithmetic operations, reading, colour naming, primary and secondary languages, etc.) it is important to establish the role of development in the acquisition of such asymmetries since most adults become familiar with such tasks and related representations (i.e., symbols) in early childhood.
